# Supplementary material for: Diurnal moths have larger hearing organs: evidence from comparative 3D morphometric study on geometrid moths
Source: PeerJ. 2025 Aug 26;13:e19834. doi: 10.7717/peerj.19834 (PMC12396209; doi:10.7717/peerj.19834)
Supplement: Supplemental Information 1 [file peerj-13-19834-s001.docx]

| **Subfamily** | **Diurnal** | **Nocturnal** | **Level of relatedness** | **Region and collecting year** |
| --- | --- | --- | --- | --- |
| Ennominae | *Sciadia tenebraria* | *Sciadia zelleraria* | Same genus | Austria (both species: 2021) |
| Ennominae | *Milionia delicatula* | *Ectropis crepuscularia* | Same tribe | Indonesia,1996 (*Milionia*), Finland,1993 (*Ectropis*) |
| Ennominae | *Venilodes woodhalli* | *Mauna ava* | Same tribe | South Africa (*V.woodhalli*: 2015; *M.ava*: 2012) |
| Ennominae |  | *Arichanna sp.* |  | India (2018) |
| Ennominae |  | *Loxaspilates sp* |  | India (2018) |
| Archiearinae | *Archiearias parthenias* |  | All Archiearinae are diurnal | Finland (2021) |
| Larentiinae | *Rheumaptera hastata* | *Rheumaptera cervinalis* | Same genus | Finland (*R. hastata*: 1996; *R. cervinalis*:1993) |
| Geometrinae | ***Dysphania percota*** | *Pingasa sp.* | Sister tribes | India (2021) |
| Oenochrominae |  | *Oenochroma vinaria* | All Oenochrominae are nocturnal | Australia (2009) |
| Desmobathrinae | *Gypsochroa renitidata* | *Conolophia conscitaria* | Same tribe | Turkey (*Gypsochroa*: 1998), Thailand (*Conolophia*: 2016) |
| Sterrhinae | *Aletis concolor* | *Problepsis ctenophora* | Same tribe | South Africa (Aletis:2023, Problepsis: 2012) |
| Epidesmiinae |  | *Abraxaphantes perampla* | Few Epidesmiinae are cathemeral (but not included in the study) | Thailand (2006) |

Table S1: The Geometridae moth species selected for this study are listed below. The species highlighted in **bold** exhibit cathemeral behavior, being active both during the day and night. Consequently, this species was excluded from all diurnal-nocturnal comparative analyses.


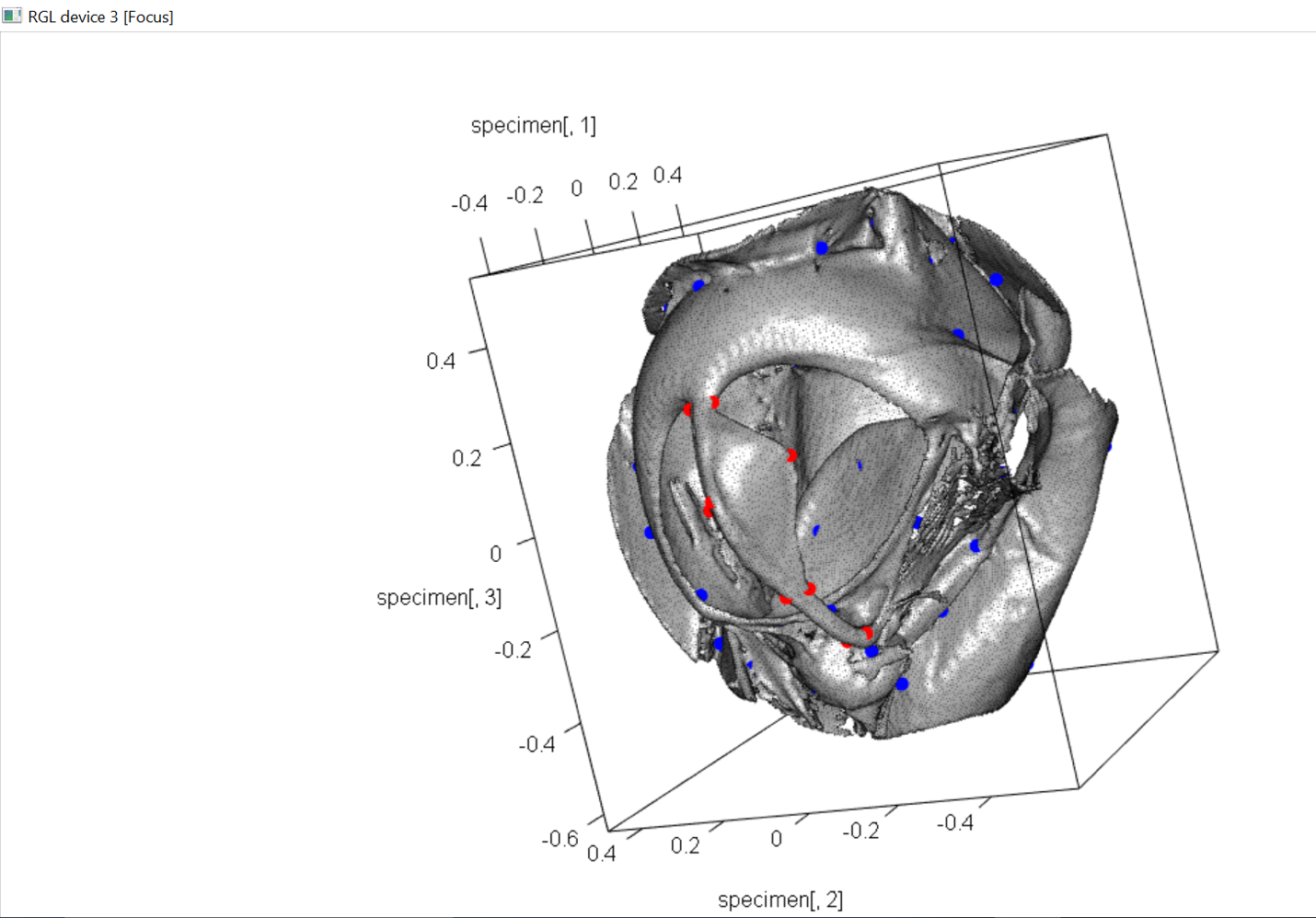


Fig. S2: A 3D model of the tympanal organ of *Loxaspilates sp.*, with ten digitized landmarks (red) on ansa and generated semi-landmarks (blue) on the tympanal organ. The red landmarks were used for 3D geometric morphometric analysis to compare the shape and size of ansa among species.
