# Supplementary material for: Diurnal moths have larger hearing organs: evidence from comparative 3D morphometric study on geometrid moths
Source: PeerJ. 2025 Aug 26;13:e19834. doi: 10.7717/peerj.19834 (PMC12396209; doi:10.7717/peerj.19834)
Supplement: Supplemental Information 4 [file peerj-13-19834-s004.docx]

The DOI link for .ply files of the tympanal organs (Right and left) of the 19 geometrid species used in our study

1. *Conolophia* sp.

<https://doi.org/10.17602/M2/M741684>

<https://doi.org/10.17602/M2/M741680>

1. Ectropis crepuscularia

<https://doi.org/10.17602/M2/M741675>

<https://doi.org/10.17602/M2/M741670>

1. Dysphania percota

<https://doi.org/10.17602/M2/M741659>

<https://doi.org/10.17602/M2/M741658>

1. Pingasa sp.

<https://doi.org/10.17602/M2/M741654>

<https://doi.org/10.17602/M2/M741651>

5. *Abraxaphantes perampla*

<https://doi.org/10.17602/M2/M741647>

<https://doi.org/10.17602/M2/M741646>

6. *Cartaletis libyssa*

<https://doi.org/10.17602/M2/M741642>

<https://doi.org/10.17602/M2/M741639>

7. *Archiearis parthenias*

<https://doi.org/10.17602/M2/M741635>

<https://doi.org/10.17602/M2/M741634>

8. *Arichanna* sp.

<https://doi.org/10.17602/M2/M741630>

<https://doi.org/10.17602/M2/M741627>

9. *Gypsochroa renitidata*

<https://doi.org/10.17602/M2/M741608>

<https://doi.org/10.17602/M2/M741605>

10. *Rheumaptera cervinalis*

<https://doi.org/10.17602/M2/M741602>

<https://doi.org/10.17602/M2/M741599>

11. *Loxaspilates sp.*

<https://doi.org/10.17602/M2/M741595>

<https://doi.org/10.17602/M2/M741594>

12. *Mauna ava*

<https://doi.org/10.17602/M2/M741590>

<https://doi.org/10.17602/M2/M741587>

13. *Oenochroma vinaria*

<https://doi.org/10.17602/M2/M741583>

<https://doi.org/10.17602/M2/M741582>

14. *Problepsis ctenophora*

<https://doi.org/10.17602/M2/M741575>

<https://doi.org/10.17602/M2/M741571>

15. *Rheumaptera hastata*

<https://doi.org/10.17602/M2/M741566>

<https://doi.org/10.17602/M2/M741563>

16. Sciadia tenebraria

<https://doi.org/10.17602/M2/M741557>

<https://doi.org/10.17602/M2/M741554>

17. Sciadia sp.

<https://doi.org/10.17602/M2/M741544>

<https://doi.org/10.17602/M2/M741541>

18. Venilodes sp.

<https://doi.org/10.17602/M2/M741537>

<https://doi.org/10.17602/M2/M741534>

19. Milionia sp.

<https://doi.org/10.17602/M2/M741529>

<https://doi.org/10.17602/M2/M741522>
